# *De novo* or Salvage? Nucleotide Availability as a Driver of Bacterial Adaptation and Virulence

**DOI:** 10.1002/mbo3.70358

**Published:** 2026-07-02

**Authors:** Riya Joshi, Alastair G. McEwan, Ulrike Kappler

**Affiliations:** ^1^ School of Chemistry and Molecular Biosciences The University of Queensland St. Lucia QLD Australia

**Keywords:** biosynthesis, *de novo* pathway, nucleotide metabolism, purine, pyrimidine, salvage pathway

## Abstract

Bacterial pathogens rely on the constant availability of purine and pyrimidine nucleotides to facilitate replication, growth, and virulence and to sustain energy metabolism and nucleotide‐based signaling. The capacity to switch between *de novo* synthesis and salvage pathways underpins much of their metabolic flexibility and also regulates access to different human body niches, where nucleobase availability varies significantly between extracellular fluids, mucosal surfaces, inflamed tissues, and intracellular compartments. However, adaptation to specific host niches can result in the loss of *de novo* nucleotide biosynthesis pathways, increasing bacterial dependence on nucleobase/nucleoside salvage. Many intracellular pathogens lack *de novo* synthesis pathways, making purine or pyrimidine salvage not an optional, but an essential process where host nucleotide reserves are critical to bacterial survival. Because of their central role in bacterial metabolism, enzymes, transporters, and regulatory networks involved in purine and pyrimidine metabolism represent potential targets for therapeutic interventions. This review summarizes the current knowledge of purine and pyrimidine metabolism in bacterial pathogens, including the abundance of these compounds in different host niches, tissue‐specific fitness strategies, and bacterial targets for further development of innovative antibacterials.

## Graphical abstract

Bacterial pathogens survive in different host environments by switching between making nucleotides by *de novo* synthesis and scavenging them from the host. This flexibility supports growth and virulence making nucleotide metabolism an attractive therapeutic target.

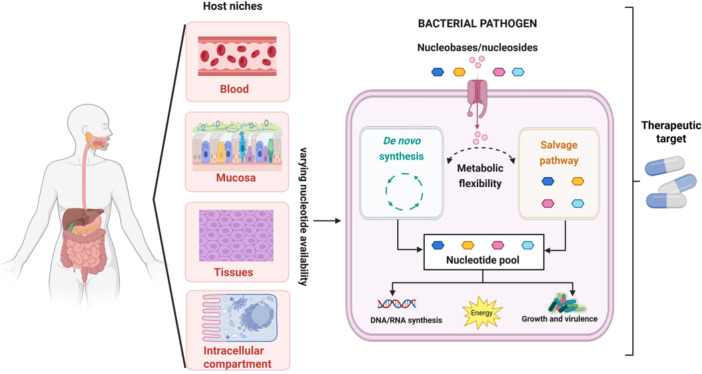

## 1 Introduction

Purine and pyrimidine nucleobases are essential components of all living cells, where they serve as building blocks of DNA and RNA, as cofactors for enzymatic reactions, components of NAD(P) and NAD(P)H, and enable energy conservation in the form of ATP and related nucleotides (King et al. 2006; Buckstein et al. 2008; Jensen et al. 2008; Nyhan 2014). Availability of nucleobase‐containing molecules is essential beyond DNA and RNA synthesis and can impact general cell metabolism, including the respiratory chain, and cell wall biosynthesis, where UTP is used to modify structural building blocks (Jensen et al. 2008; Samant et al. 2008). Nucleotide‐based molecules are also essential for regulation and signal transduction in bacteria. Secondary messengers such as cyclic di‐GMP and alarmones, such as (p)ppGpp, contribute to the regulation of cellular responses to stress, expression of virulence factors, and nutrient acquisition (Corrigan et al. 2013; Irving et al. 2021; Purcell and Tamayo 2016). As a result of the many and varied functions of nucleotides and nucleosides in living cells, maintaining a sufficient supply of these molecules is essential for cell survival (Alcantara et al. 2004; Donini et al. 2017; Liechti and Goldberg 2012; Mei et al. 1997).

Consistent with their fundamental importance, over the past several decades, research has shown a strong correlation between disruption in nucleotide metabolism and reduced bacterial pathogenicity (Clemmer et al. 2011; Ge et al. 2008; Yoshioka and Newell 2016; Wheeler et al. 2005; Ueda et al. 2009; Rodríguez‐Arce et al. 2017; Pang et al. 2012). This review summarizes the current understanding of purine and pyrimidine biosynthesis and salvage pathways with a particular focus on how they shape access of pathogenic bacteria to specific niches within a host organism, bacterial fitness, and pathogenicity.

## 2 Purine and Pyrimidine Biosynthetic Pathways and Their Impact on Bacterial Virulence

Purines and pyrimidines can be acquired by cells in two ways: by *de novo* synthesis from small‐molecule precursors or by utilization of nucleoside precursors present in the environment through salvage pathways (Chua and Fraser 2020; Nygaard 2014; Switzer and Quinn 2014).

### 2.1 *De novo* Biosynthesis Pathways

The *de novo* synthesis of nucleobases is a metabolically expensive process that requires many catalytic steps to generate the purine or pyrimidine rings from simple carbon and nitrogen molecules (Zalkin and Dixon 1992; Switzer and Quinn 2014). As a result, the *de novo* synthesis pathways are extensively regulated by a variety of mechanisms, including control of gene transcription, post‐transcriptional regulation, and feedback inhibition of enzymes (Xu et al. 2007; Matsui et al. 2001).

**Figure 1:**
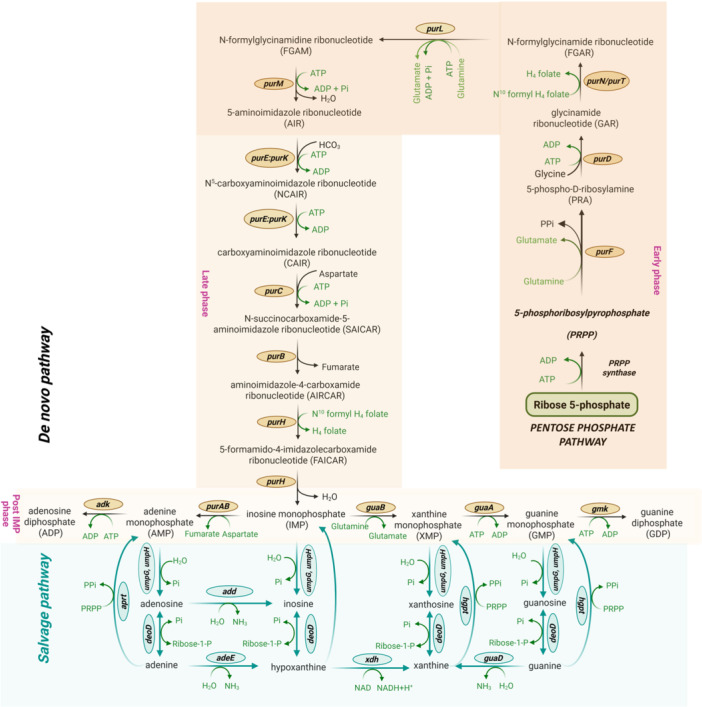
Purine metabolic pathway in bacteria. Schematic overview of the bacterial purine metabolic network, including the *de novo* biosynthesis pathway from PRPP to IMP, which is divided into two phases as indicated (orange and light orange), with the third phase representing the conversion of IMP to adenine and guanine nucleotides (yellow). The salvage route, depicted in blue, recycles purine bases and nucleosides. The schematic diagam is based on the purine metabolic pathway of *Escherichia coli*.

The *de novo* biosynthesis of purines requires the initial production of 5‐phosphoribose‐1‐pyrophosphate (PRPP) from the pentose phosphate pathway intermediate ribose 5‐phosphate (Chua and Fraser 2020; Silva et al. 2019; Engelking 2015). The reactions that then give rise to the purine ring structure form three major biosynthesis phases. Early biosynthesis leads to the formation of the first purine base, aminoimidazole ribonucleotide (AIR) (Thoden et al. 1999; Xu et al. 2007). This is followed by late biosynthesis that results in the formation of inosine monophosphate (IMP), which is the final product of *de novo* purine biosynthesis (Xu et al. 2007). Lastly, the post‐IMP stage mediates the conversion of IMP to the more common cellular purine bases such as adenosine monophosphate (AMP) and guanosine monophosphate (GMP) (Chen et al. 1992; Matsui et al. 2001; Tsai et al. 2014). Nucleoside monophosphate and diphosphate kinases then convert AMP and GMP to adenosine/guanosine diphosphates (ADP and GDP) and adenosine/guanosine triphosphates (ATP and GTP), respectively (Zalkin and Dixon 1992; Kappock et al. 2000; Jensen et al. 2008) (Figure 1). A detailed review of the enzymology of this pathway can be found in (Chua and Fraser 2020).

**Figure 2:**
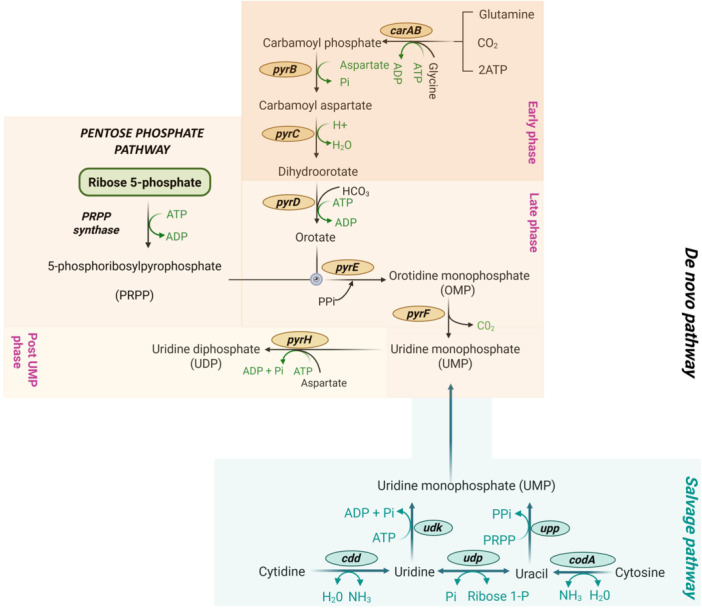
Pyrimidine metabolic pathway. Schematic representation of the bacterial pyrimidine metabolic network, including the *de novo* biosynthesis pathway from glutamine and bicarbonate to UMP, indicated as early (orange) and late phase (light orange). The post UMP phase involves the conversion of UMP to cytosine, uracil, and thymine nucleotides (yellow). The salvage route, depicted in blue, recycles pyrimidine bases and nucleosides. The schematic diagram is based on the pyrimidine metabolic pathway of *Escherichia coli*.

In contrast to the complex pathway of purine biosynthesis, the *de novo* production of pyrimidines requires only six enzymatic steps, where, using glutamine, bicarbonate, and aspartate, the pyrimidine ring is assembled and then attached to PRPP, yielding uridine monophosphate (UMP) (Donini et al. 2017; Jensen et al. 2008). However, biosynthesis follows similar phases to what we described above for purines: During early biosynthesis, the reactions of aspartate transcarbamylase, carbamoyl phosphate synthetase, and dihydroorotase produce dihydroorotate, a compound that incorporates the basic pyrimidine ring structure. In the late biosynthesis phase, dihydroorotate dehydrogenase oxidizes dihydroorotate to orotate, followed by the PRPP requiring conversion of orotate to UMP by UMP synthase. Lastly, in the post‐UMP phase, UMP is converted to cytidine triphosphate (CTP) and thymidine triphosphate (TTP) and their deoxy‐forms, which are utilized for RNA and DNA synthesis and other cellular processes (Garavaglia et al. 2012) (Figure 2). Additionally, in *de novo* nucleotide biosynthesis, folate‐derived one‐carbon units play an essential role in both purine and thymidylate production (Li et al. 2017; Goldstein and Proctor 2008; Stover 2009; Yaeger et al. 2025). Specifically, 10‐formyl‐tetrahydrofolate donates carbon groups to the GAR and AICAR transformylase steps of purine biosynthesis, while 5,10‐methylene‐tetrahydrofolate is required by thymidylate synthase for the conversion of dUMP to dTMP (Li et al. 2017; Goldstein and Proctor 2008; Stover 2009; Yaeger et al. 2025).

### 2.2 Salvage Pathways

Purine and pyrimidine salvage pathways mediate the uptake of external nucleobases or nucleosides followed by conversion into the corresponding nucleotides, allowing the bacteria to minimize energy consumption and enabling utilization of nucleobases and derivatives available in their environment (Nyhan 2014, G. Ducati et al. 2011, D. Villela et al. 2011; Yuan et al. 2019).

#### 2.2.1 Transporters

**Figure 3:**
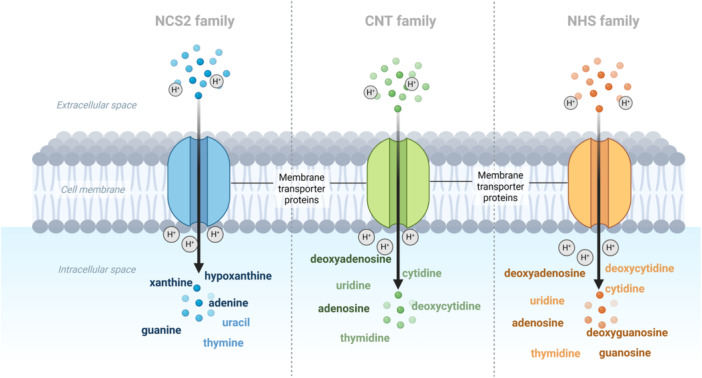
Nucleobase and nucleoside transporters in bacteria. The picture depicts major transporter families: nucleobase cation symporter 2 (NCS2), concentrative nucleoside transporter (CNT), and nucleoside H^+^ symporter (NHS) which mediate uptake of purine and pyrimidine nucleobases and nucleosides in bacterial pathogen.

Utilization of purine and pyrimidine nucleobases or nucleosides present in the extracellular environment requires specific transporters (Webb and Hosie 2006; Wang et al. 2021; Patching et al. 2005; Papakostas et al. 2013; Lu et al. 2011; King et al. 2006). Both ATP‐dependent primary transporters and secondary transporters linked to ion gradients have been shown to be involved (Zhang 2025). In bacteria, the transporters that mediate the uptake of purine or pyrimidine compounds belong to three major families: the Nucleobase‐Cation Symporter‐2 (NCS2) transporters (Botou et al. 2018), Concentrative Nucleoside Transporters (CNT), and Nucleoside proton H^+^ Symporters (NHS) (Munch‐Petersen et al. 1979) (Figure 3). Transporters from all three families mediate ion/proton gradient‐dependent active transport of their substrates across the plasma membrane of bacterial cells.

With over 2000 putative members identified across all important taxa of bacteria or also outside of bacteria, NCS2 family transporters are distinguished by their evolutionary ubiquity across all domains of life (Botou et al. 2018). NCS2 family transporters have been shown to transport both pyrimidine (uracil and thymine) and purine (adenine, guanine, xanthine, hypoxanthine) nucleobases (Krypotou et al. 2014; Botou et al. 2018; Cecchetto et al. 2004), and the first identified representative of this group was UapA from *Aspergillus nidulans* (Diallinas and Scazzocchio 1989; Cecchetto et al. 2004; Gorfinkiel et al. 1993). In bacteria, the first characterized members of the NCS2 family were the uracil and xanthine transporters, UraA and XanQ, from *Escherichia coli*, after which PyrP was reported to transport uracil in *Bacillus subtilis* (Lu et al. 2011; Krypotou et al. 2014; Karena and Frillingos 2011; Botou et al. 2018; Andersen et al. 1995). The UraA transporter from *E. coli* was the first representative of this group for which a high‐resolution crystal structure was solved (Andersen et al. 1995; Lu et al. 2011). It showed that the protein consists of two domains, a ‘gate’ (TMs 5‐7, 12‐14) and a “core”’ domain, and that substrate binding is exclusively mediated by the core domain (TMs, 1‐4, 8‐11) with two glutamate residues (Glu241 and Glu 290) that hydrogen‐bond to the substrate molecule (Karena and Frillingos 2011; Sioupouli et al. 2017; Diallinas and Scazzocchio 1989; Andersen et al. 1995; Lu et al. 2011; Botou et al. 2018).

Other NAT/NCS2 family nucleobase transporters have been shown to contribute to purine transport in *E. coli* and include AzgA, which comprises adenine, guanine, and hypoxanthine transporters, and the XanP and XanQ high‐affinity xanthine and hypoxanthine transporters (Krypotou et al. 2014; Botou et al. 2018; Karena and Frillingos 2011). According to phylogenetic and functional investigations, the NAT/NCS2 family is divided into three primary clusters based on substrate specificity. Cluster I comprises uracil and uracil‐analogue transporters like *E. coli* UraA and *B. subtilis* PyrP. The xanthine and uric‐acid transporters in Cluster II, such as *Aspergillus nidulans* UapA and *E. coli* XanQ/XanP, have conserved residues in transmembrane segments 3, 8, and 10, which have been shown to create the purine‐binding pocket and regulate base selectivity (Karena and Frillingos 2011; Tatsaki et al. 2021; Diallinas and Scazzocchio 1989; Gorfinkiel et al. 1993). The transport of adenine, guanine, and hypoxanthine is mediated by Cluster III, the AzgA‐type group, which initially was identified in fungi and then discovered in bacteria via homologues like *E. coli* YicE and YgfO (Cecchetto et al. 2004; Krypotou et al. 2014).

In addition to NCS2 family transporters, members of the concentrative nucleoside transporters (CNT) and the nucleoside:H^+^ symporter (NHS) family also contribute to purine and pyrimidine transport and have been characterized in both *E. coli* and *B. subtilis* (Young et al. 2013; Wang et al. 2021; Patching et al. 2005). Unlike the NCS2 family transporters, CNT and NHS family proteins predominantly transport nucleosides (Lu et al. 2011; Karena and Frillingos 2011; Andersen et al. 1995). The CNT family transporters in *E. coli* include the extensively studied NupC transporter, which has wide selectivity for purine and pyrimidine nucleosides, including adenosine, cytidine, thymidine, and uridine, along with their deoxy‐derivatives, but does not transport guanosine or inosine (Saxild et al. 1996; Rodríguez‐Arce et al. 2017; Kriegeskorte et al. 2014). NupC also transports pyrimidine nucleosides and their deoxy‐derivatives in *B. subtilis* (Saxild et al. 1996). NupG, another well‐studied nucleoside transporter in bacteria, mediates the transport of all purine and pyrimidine nucleosides. NupG is a representative of the NHS family, a subgroup of the Major Facilitator Superfamily (MFS) (Wang et al. 2021), and is thus, despite the similar name, structurally unrelated to NupC. NupG plays a vital role in the uptake of purine and pyrimidine nucleosides in both *E. coli* and *B. subtilis*. Although NupC and NupG are the main transporters for nucleoside uptake in *E. coli* and *B. subtilis*, mutants lacking both of these genes only retain some capacity for purine and pyrimidine nucleoside uptake, suggesting the presence of additional, currently unidentified transporters (Saxild et al. 1996). One such transporter is the ATP‐binding cassette (ABC) transporter complex NupNOPQ from *B. subtilis*, which has been shown to mediate guanosine nucleoside uptake, underscoring the complexity and redundancy of nucleoside transport systems in bacteria (Belitsky and Sonenshein 2011). Recent research has revealed that, in addition to transporting purine and pyrimidine nucleobases, NupC and NupG homologs from *E. coli*, *Klebsiella pneumoniae*, and *Citrobacter freundii* can catalyse the high‐affinity uptake of gemcitabine, a fluorinated cytidine analog used in chemotherapy, expanding the substrate range of bacterial nucleoside transporters (Iosifidou et al. 2024).

The evolutionary diversity and high level of purine and pyrimidine transporter redundancy in bacterial pathogens highlight the vital role that nucleobase/nucleoside transport plays in cellular adaptation and survival, and several studies suggest the existence of additional, uncharacterized transport systems in several bacteria (Kennelly and Prindle 2024; Tatsaki et al. 2021; Weng et al. 2023; Baldwin et al. 1999; Saier 2000; Cecchetto et al. 2004; Webb and Hosie 2006; King et al. 2006). Transporters are not only uptake systems but key determinants of metabolic flexibility, contributing to bacterial fitness, persistence, and virulence.

#### 2.2.2 Salvage Enzymes

Following uptake into the cell via transporters, nucleobases and nucleosides undergo transformations to ensure a sufficient supply of all required purines, pyrimidines, and their derivatives. Purine nucleobases such as hypoxanthine, guanine, and adenine can be converted into the respective monophosphates IMP, GMP, and AMP by hypoxanthine/guanine/adenine phosphoribosyltransferases that use PRPP as a co‐substrate and are encoded by the *hpt, xpt, gpt*, and *apt* genes, respectively (Wheeler 1990; Duckworth et al. 2006; Becerra and Lazcano 1998; Taylor et al. 2002; Nilsson and Lauridsen 1992; Levine and Taylor 1981; Keough et al. 2009; Doyle et al. 1998) (Figure 1). If nucleosides such as guanosine, adenosine, and inosine have been taken up or formed, they can be directly converted into nucleotide monophosphates via the reversible reaction of inosine/guanosine kinase (encoded by *gsk*). Alternatively, nucleosides taken up from the environment can be broken down by purine‐nucleoside phosphorylase (encoded by *deoD)* into the respective nucleobases and ribose‐1‐phosphate to meet cellular nucleobase requirements. The nucleobases can then potentially be recycled back to nucleosides and nucleotides by purine phosphoribosyltransferases (Xi et al. 2000; del Arco and Fernández‐Lucas 2017; Levine and Taylor 1981; Becerra and Lazcano 1998; Nygaard 1993), while ribose‐1‐phospate can be funnelled into the Pentose Phosphate Pathway for energy generation.

Pyrimidine salvage uses a similar set of enzymes that mediate nucleobase or nucleoside conversions. Imported uracil can be directly converted to UMP by the uracil phosphoribosyltransferase (encoded by *upp*), which uses PRPP as a co‐substrate (Andersen et al. 1992; Silva et al. 2019). Additionally, uracil may be transformed to uridine via uridine phosphorylase (encoded by *udp*), and then further phosphorylated to UMP by uridine kinase (encoding gene: *udk*) (Wheeler 1990; Martinussen et al. 1994; Balestri et al. 2007; Tomoike et al. 2015). Externally acquired cytosine can be deaminated to uracil by cytosine deaminase (encoded by *codA*), allowing it to enter the salvage reactions outlined above (West TP 1982; Kohila et al. 2012). If nucleosides such as uridine, cytosine, or thymidine have been taken up, uridine kinase and thymidine kinase can phosphorylate these nucleosides to their corresponding monophosphates (UMP, CMP, and dTMP), with uridine kinase converting both uridine and cytidine (Markaryan et al. 2001; Balestri et al. 2007; Arvind et al. 2013; Rodríguez‐Arce et al. 2017; Kriegeskorte et al. 2014; West TP 1982; Kohila et al. 2012). Beyond converting nucleotides and nucleosides acquired from the environment, nucleobase salvage mechanisms are essential to bacterial physiology as they support recycling of cellular nucleosides and nucleobases into nucleotides. By phosphorylating nucleotides, nucleoside and nucleotide kinases generate nucleoside triphosphates that facilitate energy metabolism and generate nucleotide pools required for DNA and RNA synthesis (Corrigan et al. 2013; Fontaine et al. 2018; Liu et al. 2020; Bebenek et al. 1992; Kunz et al. 1994; Mathews 2006).

In addition to the salvage of nucleobases and nucleosides, there is evidence that some bacteria are also able to acquire intermediates of nucleotide biosynthesis from the extracellular environment and channel them into the *de novo* synthesis pathways. The best‐characterised example is the pyrimidine synthesis intermediate orotate, which can be taken up by bacteria such as *E. coli* and *Salmonella enterica* via the C4‐dicarboxylate transporter DctA (Amin et al. 2022; Baker et al. 1996). Once internalised, orotate can be metabolised by orotate phosphoribosyltransferase to form OMP and subsequently UMP, thereby allowing cells to bypass upstream steps in pyrimidine biosynthesis and restore nucleotide pools (Amin et al. 2022; Baker et al. 1996). Purine *de novo* biosynthesis pathway intermediates such as AICA (4‐aminoimidazole‐5‐carboxamide), its nucleoside AICAr (AICA riboside), and AICAR (5‐aminoimidazole‐4‐carboxamide ribonucleotide) have also been reported to rescue purine‐auxotrophic mutants in *E. coli* and *S. enterica* that carry mutations in early *de novo* biosynthesis genes (Daignan‐Fornier and Pinson 2012; Bazurto and Downs 2014). Following uptake, AICA, AICAr and AICAR are converted to IMP, restoring purine synthesis. However, compared to classical salvage pathways, the uptake of biosynthetic intermediates remains poorly understood and the extent to which other intermediates can be taken up and metabolised in a similar manner requires further investigation (Daignan‐Fornier and Pinson 2012; Bazurto and Downs 2014).

In summary, while the reactions that make up the *de novo* and salvage purine and pyrimidine pathways are well characterized, not all known reactions are found in all bacteria, and consequently, there is a wide variety of different pathway configurations. For example, while *E. coli* and *Pseudomonas aeruginosa* possess a complete complement of purine and pyrimidine *de novo* and salvage pathways, several host‐adapted pathogens such as *Haemophilus influenzae*, *Helicobacter pylori* and *Neisseria meningitidis* miss either key reactions of, for example, *de novo* biosynthesis pathways, or entire pathways (Schilling and Palsson 2000; Wong and Akerley 2012; Liechti and Goldberg 2012; Liechti and Goldberg 2013; Jyssum and Jyssum 1979). This variability reflects how bacteria adapt to nutrient availability in their respective environments, resulting in unique, species ‐specific dependencies on purine or pyrimidine sources that can be used to develop targeted therapeutic techniques.

##### 2.2.2.1 Regulation of Purine and Pyrimidine (Salvage) Pathways

Regulation of nucleobase biosynthesis has been studied in various bacteria, and regulatory mechanisms have been identified for both catabolic and biosynthetic mechanisms (Grove 2025; Gore and West 2025; Gots 1971; Saxild and Nygaard 1991; He et al. 1992; Weng et al. 1995). The regulatory patterns of purine metabolism are complex, as they not only concern the supply of purines but also interface with key regulatory circuits that rely on guanine‐based messenger molecules such as cyclic‐di‐GMP and guanosine pentaphosphate (Grove 2025). Cyclic‐di‐GMP is the essential second messenger that controls the transition from motile to sessile states, enabling biofilm formation, surface adhesion, and persistence while suppressing motility (Grove 2025; Hall and Lee 2018). It promotes adaptability to host settings by regulating the formation of extracellular polysaccharides and the expression of adhesins, and is linked to both greater antimicrobial tolerance and chronic infection (Cotter and Stibitz 2007; Monds et al. 2010; Srivastava and Waters 2012; Kennelly et al. 2024; Gupta et al. 2015). As regulation of purine metabolism has been the subject of several recent and comprehensive reviews, we will only provide a brief overview here (Grove 2025; Ayoub et al. 2024; Stevens et al. 2025; Pedley and Benkovic 2017; Tang et al. 2019).

A well‐studied regulator of purine biosynthesis in many Gamma‐Proteobacteria is PurR, which binds accumulating hypoxanthine or guanine and, in response, represses enzymes for the *de novo* synthesis of IMP (Levine and Taylor 1981; He et al. 1992; Sause et al. 2019; Goncheva et al. 2019; Anderson et al. 2022; Xiao et al. 2023). In *E. coli*, the PurR regulator controls its own expression (Grove 2025; Rolfes and Zalkin 1990); however, this trait is not conserved in all bacteria that harbour PurR homologues. The PurR binding site needed for autoregulation is absent in *Vibrio cholerae* and *Pasteurellaceae*, including *H. influenzae* (Ravcheev et al. 2002). In *B. subtilis*, a regulator designated PurR also regulates IMP synthesis, but is structurally unrelated to the *E. coli* protein and is activated by binding of PRPP, the precursor for purine biosynthesis (Rappu et al. 2003).

The ‘alarmone’ (p)ppGpp leads to a downregulation of purine biosynthesis in both Gram‐negative and Gram‐positive bacteria during starvation to increase survival and avoid growth defects resulting from reduced levels of PRPP for biosynthesis (Battesti and Bouveret 2009; Taylor et al. 2002; Gupta et al. 2015; Irving et al. 2021; Anderson et al. 2022). In *B. subtilis*, (p)ppGpp additionally inhibits purine salvage via modulation of phosphoribosyltransferase activity (Anderson et al. 2022; Anderson et al. 2020). Control of guanine levels is also linked to cyclic‐di‐GMP levels and thus interacts with another major regulatory circuit. The complexity of regulatory networks associated with purine homeostasis is further illustrated by studies in various bacteria that indicate cross‐regulation of purine biosynthesis by the RbsR ribose metabolism regulator in *E. coli*, and moonlighting of PurR as a regulator of virulence in *Staphylococcus aureus* and *Yersinia pestis* (Sause et al. 2019; Xiao et al. 2023; Shimada et al. 2013). Riboswitches form another layer of purine‐based regulation (Kirchner and Schneider 2017; Kofoed et al. 2016). Progress in understanding the sensing of purine levels has been made through the recent identification of a purine‐sensing domain that is found in various sensor proteins, following on from an earlier identification of a chemosensor that enables motility towards purine sources (Fernández et al. 2016; Monteagudo‐Cascales et al. 2024).

Regulation of pyrimidine production relies on regulators such as PyrR in response to UMP levels in Gram‐positive bacteria (Fields and Switzer 2007; Tomchick et al. 1998). In contrast, in Gram‐negative bacteria, *pyr* genes are more commonly regulated by mechanisms that cause premature termination or changes in transcription start sites in response to changes in nucleotide abundance (Turnbough and Switzer 2008). The regulation occurs as a result of ‘reiterative transcription’, also known as transcription slippage, and relies on elements in the promoter regions of the *pyr* genes (Shin et al. 2020; Turnbough and Switzer 2008).

As indicated above, these regulatory systems are tightly connected to nucleotide availability, and this allows bacteria to adapt to purine‐ and pyrimidine‐limited conditions including within the host, where substrates for salvage processes. In pathogens such as *S. aureus* and *Yersinia pestis*, PurR repression is released under purine limitation, resulting in a rapid upregulation of purine biosynthesis genes (Sause et al. 2019; Goncheva et al. 2020; Xiao et al. 2023). Simultaneously, the host‐induced nutrient stress triggers the (p)ppGpp‐mediated stringent response, which regulates nucleotide metabolism by decreasing *de novo* nucleobase synthesis, keeping a balance between nucleotide requirements and availability of energy sources (Gupta et al. 2015; Irving et al. 2021; Anderson et al. 2022; Kennelly et al. 2024). This supports survival and persistence in nutrient‐restricted niches, for example, for *Mycobacterium tuberculosis* in granulomas (Klinkenberg et al. 2010). Similar processes govern pyrimidine biosynthesis regulation, via mechanisms such as PyrR and reiterative transcription, also provides for fine‐tuning of UMP production in response to changes in intracellular nucleotide levels (Tomchick et al. 1998; Fields and Switzer 2007; Turnbough and Switzer 2008).

## 3 Availability of Purines and Pyrimidines in Host Niches and Bacterial Adaptation

### 3.1 Host Niches and Their Profiles

To thrive in a host during infection, bacterial pathogens must adapt their metabolic processes to suit the environmental abundance of substrate molecules, including purines and pyrimidines, to what is available in the host niche (Passalacqua et al. 2016; Traut 1994; Kilstrup et al. 2005; Kelley and Andersson 2014; Fumagalli et al. 2017; Goncheva et al. 2022; Narayanan et al. 2022) (Table 1). For a long time, it was assumed that differentiating host cells would cover most of their purine and pyrimidine demand by *de novo* synthesis, while differentiated host cells would use the salvage pathway instead (Tran et al. 2024; Villa et al. 2019; Ali and Ben‐Sahra 2023; Sokolov and Sullivan 2024). A recent study, however, showed that *de novo* purine biosynthesis takes place in most differentiated tissues at different, tissue‐specific levels (Tran et al. 2024). Pyrimidine biosynthesis may follow a similar pattern, creating tissue‐specific variation in the availability of purines and pyrimidines.

**Table 1:** Nucleotide Biosynthesis Strategies Across Major Pathogenic Bacteria. The Table Shows *de novo* Purine/Pyrimidine Synthesis, Salvage Pathways Across Different Taxonomic Groups.

Taxonomic group	Pathogen	Gram	Main niche(s)	*De novo* Pur/pyr	*De novo* essentiality	Salvage Pur/Pyr	Salvage essentiality	Key insight	References
Bacilli	*Staphylococcus aureus*	+	Abscess, systemic	✓/✓	Yes	✓/✓	Essential	*De novo* supports invasion, while salvage enhances fitness in nucleotide‐rich abscesses.	(Goncheva et al. (2020); Connolly et al. (2017))
	*Streptococcus pneumoniae*	+	Airway, blood	✓/✓	Yes	✓/✓	Moderate	*De novo* is required for in vivo colonisation and infection	(Carvalho et al. (2018); Polissi et al. (1998))
	*Bacillus anthracis*	+	Bloodstream	✓/✓	Yes	✓/✓	Not sufficient	Serum is nucleotide‐poor, so salvage cannot rescue auxotrophy.	(Ivanovics et al. (1968); Jenkins et al. (2011); Samant et al. (2008))
	*Listeria monocytogenes*	+	Cytosol	✓/✓	Fitness, not absolute	✓/✓	Strongly supportive	Salvage compensates *de novo* loss.	(Klarsfeld et al. (1994); Marquis et al. (1993); Narayanan et al. (2022))
Actinomycetes	*Mycobacterium tuberculosis*	+	Phagosome	✓/✓	Strictly essential	✓/✗	Not sufficient	Phagosomes are nucleotide‐poor, creating a strong dependence on *de novo* synthesis.	(Lamprecht et al. (2025); Sassetti and Rubin (2003); Wheeler (1990))
α ‐proteobacteria	*Brucella spp*.	—	Macrophage vacuole	✓/✓	Yes/strong	✓/✓	Not sufficient	The vacuole is nutrient‐restricted, so salvage is ineffective despite being present.	(Alcantara et al. (2004); Crawford et al. (1996); Drazek et al. (1995))
	*Rickettsia spp*.	—	Host cytosol	✗/✗	N/A	✓/✓	Essential (transport)	ATP/NTP parasites bypass both *de novo* synthesis and classical salvage.	(Radulovic et al. (2002); Zomorodipour and Andersson (1999))
β‐ proteobacteria	*Neisseria meningitidis*	—	Blood, CSF	✓/✓	Strictly essential	✓/✗	Not sufficient	Extremely poor nucleotide availability‐ minimal salvage capacity and strict *de novo* dependence.	(Jyssum and Jyssum (1979))
γ‐ proteobacteria	*Escherichia coli*	—	Blood, urinary tract, gut	✓/✓	Yes	✓/✓	Moderate	*De novo* is essential in blood, but utilizes nucleotide salvage pathways to thrive in urine and gut	(Samant et al. (2008); Shaffer et al. (2017); Vogel‐Scheel et al. (2010))
	*Acinetobacter baumannii*	—	Lung, bloodstream	✓/✓	Yes	✓/✓	Weak	*De novo* purine biosynthesis contributes to virulence	(Palmer et al. (2026); Peng et al. (2024); Wang et al. (2014b))
	*Pseudomonas aeruginosa*	—	CF lung	✓/✓	Strong in chronic lung	✓/✓	Weak	*De novo* synthesis is crucial for in vivo and in in vitro survival	(Niazy et al. (2022); Al Ahmar et al. (2019))
	*Francisella tularensis*	—	Cytosol	✓/✓	Partial, niche‐dependent	✓/✓	Moderate	Relies on both *de novo* and salvage pathway	(Horzempa et al. (2010); Pechous et al. (2006); Quarry et al. (2007))
	*Salmonella enterica*	—	Gut, blood	✓/✓	Yes in blood; reduced in gut	✓/✓	Moderate (gut)	Gut allows salvage compensation, while bloodstream requires *de novo* synthesis.	(Jelsbak et al. (2016); Morgan et al. (2004); Samant et al. (2008))
	*Haemophilus influenzae*	—	Respiratory tract, blood	✓/✗	Pur: Yes; Pyr: N/A	✓/✓	Essential (Pyr salvage)	Pyrimidine auxotrophy drives strict dependence on salvage, while purine *de novo* is upregulated in vivo.	(Gawronski et al. (2009); Wong and Akerley (2012))
ε‐ proteobacteria	*Helicobacter pylori*	—	Gastric mucosa	✗/✓	Pur: N/A; Pyr: Yes	✓/✓	Essential (Pur)	Evolutionary loss of *de novo* purine synthesis leads to complete reliance on purine salvage in a nutrient‐rich gastric niche.	(Liechti and Goldberg (2012); Liechti and Goldberg (2013); Mendz et al. (1994); Miller et al. (2012))
Chlamydiia	*Chlamydia spp*.	—	Intracellular inclusion	✗/✗	N/A	✓/✓	Essential (transport)	Extreme genome reduction, rely entirely on host NTP import.	(Bastidas et al. (2013); Herweg and Rudel (2016); Zomorodipour and Andersson (1999))
Mollicutes	*Mycoplasma spp*.	none	Respiratory mucosa	✗/✗	N/A	✓/✓	Essential	Host mucosa provides nucleotides, producing salvage‐dominated metabolism.	(Mitchell and Finch (1977); Schimmelpfeng et al. (1980))

Purine and pyrimidine concentrations have mostly been studied for extracellular fluids such as plasma, cerebrospinal fluid (CSF), airway surface liquid (ASL), and urine, where their availability, especially for free nucleobases and deoxyribonucleosides, was generally found to be very restricted (Traut 1994; Thane Eells and Spector 1983; Harkness et al. 1980; Harkness and Lund 1983). However, not all studies have reported the same analytes or used similar analytic techniques, which makes detailed comparisons difficult to achieve.

In CSF, the only identifiable nucleobases were hypoxanthine and xanthine, at concentrations of ~2.5 µM each, while guanine, thymine, cytosine, uracil, and adenine were all below detection limits (< 0.1‐0.2 µM) (Traut 1994; Thane Eells and Spector 1983; Harkness et al. 1980; Harkness and Lund 1983). Blood plasma was also low in nucleobases, with concentrations around ~0.4−0.6 µM for xanthine and hypoxanthine. Low levels (≤ 0.2 µM) of the nucleosides cytidine, inosine, and adenosine were also present in plasma samples. The only nucleoside with increased availability in both plasma and CSF was uridine, which was consistently reported at concentrations of 2−3 µM (Thane Eells and Spector 1983). Nucleotide availability in respiratory tract airway surface liquid (ASL) was even lower, with ATP concentrations ranging from 0.1 to 0.3 μM and UTP levels between 20 and 50 nM (Esther et al. 2011). Nucleobases and nucleosides were not reported; however, a study from 2018 reported detection, but not quantification, of most purine and pyrimidine nucleobases, nucleosides, and nucleotides in nasal and bronchial airway fluids (Farne et al. 2018). A study that analysed exhaled breath condensate (EBC) indicated that healthy non‐smokers had an average EBC adenosine content of 1.7 ± 1.5 μM, giving a baseline for extracellular purine levels in the airway milieu (Esther et al. 2011). In the urine of healthy individuals, low micromolar amounts of purine and pyrimidine metabolites were detected, with pyrimidine nucleosides ranging from 0.1 to 1 µM and the purine nucleobases hypoxanthine and xanthine found at around 1−2 µM (Thane Eells and Spector 1983; Traut 1994; Harkness and Lund 1983). In the human gastrointestinal system, purine and pyrimidine nucleoside concentrations were found to be below the detectable levels (< 5 μM) in the colon and small intestinal contents (Vogels and Van Der Drift 1976; Vogel‐Scheel et al. 2010). In summary, for extracellular body niches where data are available, purine and pyrimidine concentrations are generally in the low micromolar range and are frequently even below the detection limit.

In contrast to the extracellular body niches, the purine and pyrimidine availability is high inside human cells, and many bacterial pathogens are able to colonize these environments (Traut 1994; Liechti and Goldberg 2013; Goncheva et al. 2020; Fuchs et al. 2012; Fu et al. 2025). Typical intracellular nucleotide levels have been reported as ATP ~ 3152 μM, GTP ~ 468 μM, UTP ~ 567 μM, and CTP ~ 278 μM for mammalian cells; however, it should be noted that these concentrations can differ depending on the cellular activation status and tissue the cells originate from (Traut 1994). As an example, in dividing mammalian cells that have a high demand for nucleotides to support DNA synthesis, intracellular concentrations of nucleotides were reported to be approximately 37 μM dTTP, 29 μM dCTP, 24 μM dATP, 5.2 μM dGTP, and ~0.2 μM dUTP (Harkness et al. 1980; Harkness and Lund 1983; Bronk et al. 1991; Traut 1994; Fu et al. 2025). Information about the tissue‐specific concentrations of intracellular nucleotides could shed light on the conditions that intracellular pathogens face in vivo, but it is currently not readily available.

Extracellular purine and pyrimidine concentrations also vary as a result of metabolic demand, inflammation, and proliferation requirements (Kohnken et al. 2015). Increased concentrations of purine metabolites such as hypoxanthine and xanthine in CSF have been linked to conditions such as dementia and brain damage, and are thought to indicate higher oxidative stress and nucleotide breakdown (Tohgi et al. 1993). Increased uric acid, hypoxanthine, and uridine levels in blood and serum have been connected to metabolic, inflammatory, and neoplastic disorders (Stover et al. 1997; Yang et al. 2024; Toledo‐Ibelles et al. 2021; Wolf et al. 1999). For example, ischemic stroke patients are reported to have elevated serum hypoxanthine levels, which have been associated with neuroinflammation and disruption of the blood‐brain barrier (Pang et al. 2012; Toledo‐Ibelles et al. 2021; Tran et al. 2024). Colitis has also been linked to elevated hypoxanthine levels, suggesting that it plays a role in intestinal inflammation, while elevated uridine levels have been associated with diabetes, obesity, and alcoholic liver disease (Liu et al. 2025; Yang et al. 2024).

Chronic respiratory conditions such as chronic obstructive pulmonary disease (COPD) can increase purine availability in the lower airways in the airway surface liquid (ASL). In exhaled breath condensate (EBC), adenosine levels of 3.2 ± 2.7 μM were reported for COPD patients, nearly double the level (1.7 ± 1.5 μM) detected in healthy individuals (Esther et al. 2011; Staples et al. 2016). Although no measurements of nucleotide concentrations were reported, infection with *Streptococcus pneumoniae* caused an increased availability of branched chain amino acids in the nasopharynx (Green et al. 2023). It would be interesting to determine whether these metabolic changes seen are specific to the infecting bacteria or a general response to bacterial infection.

Another potential source of purine and pyrimidine nucleotides in extracellular environments, particularly during infection, is extracellular DNA and RNA (Dengler et al. 2015; Goodman and Bakaletz 2022; Thomas et al. 2008; Vilain et al. 2009). Although difficult to quantify, these nucleic acids can accumulate through host cell lysis, microbial turnover, and the release of neutrophil extracellular traps (NETs), which are composed largely of chromatin (Miller‐Ocuin et al. 2019; Rada 2019). Several pathogens, including *S. aureus, E. coli, Streptococcus pyogenes*, and *P. aeruginosa*, secrete extracellular DNAses that enable the breakdown and utilisation of NET‐derived DNA as a source of carbon, nitrogen, and phosphorus (Deng et al. 2022; Dengler et al. 2015; Cherny Kathryn and Sauer 2019; Huang et al. 2022). This highlights a dual role for NETs during infection, serving both host defence and as a nutrient source that can be co‐opted by bacterial pathogens. Collectively, DNAse‐mediated degradation of extracellular nucleic acids is a strategy that links nutrient scavenging and adaptation to nucleotide‐limited extracellular niches.

### 3.2 *De novo* or Salvage? Use of Nucleotide Acquisition and Synthesis Pathways as a Metabolic Signature of Pathogen Adaptation and Virulence

The majority of bacterial pathogens contain both a fully functioning *de novo* synthesis and a salvage pathway for the production or acquisition of purine and pyrimidine nucleotides (Aiba and Mizobuchi 1989; Flannigan et al. 1990; Samant et al. 2008; Ali et al. 2013; Gallie et al. 2015; Li et al. 2015; Andersen‐Civil et al. 2018; Goncheva et al. 2020). This metabolic adaptability allows the bacteria to survive in a broad range of host habitats (Holmes et al. 2023; O'Callaghan et al. 1988; Crawford et al. 1996; Xiong et al. 2022; Connolly et al. 2017). These ‘dual‐pathway’ organisms frequently show niche‐specific dependence on one pathway over the other, with *de novo* biosynthesis indispensable in nucleotide‐poor environments such as blood, cerebrospinal fluid (CSF), and other extracellular fluids in the host body, and salvage being prevalent in niches rich in host‐derived nucleotides (Ali et al. 2013; Yan et al. 2017; Ma et al. 2018; Kennelly et al. 2024; Connolly et al. 2017).

Disrupting *de novo* nucleotide synthesis significantly reduces the potential for bacterial infection (Samant et al. 2008; Kriegeskorte et al. 2014; Kofoed et al. 2016; Yang et al. 2017; Andersen‐Civil et al. 2018; Goncheva et al. 2020; Rossi et al. 2022). Mutations in genes such as *purF, purD, purM*, and *purN* that affect early purine biosynthesis, impair systemic dissemination, intracellular replication, or survival in contact with immune cells in *E. coli, S. typhimurium*, *S. enterica, S. aureus, Brucella abortus, Brucella melitensis, Listeria monocytogenes*, *Mycobacterium tuberculosis*, and *Francisella tularensis* (Quarry et al. 2007; Shi et al. 2014; Truong et al. 2015; Li et al. 2018; Kinsinger et al. 2005; Pechous et al. 2006; Liang et al. 2021; Narayanan et al. 2022; Drazek et al. 1995; Crawford et al. 1996; Jackson et al. 1999). In a similar manner, the deletion of genes of the post‐IMP purine biosynthesis phase (*purA, purB, guaA, guaB*) is consistently associated with reduced tissue colonization, lower bacterial burdens in serum or blood, and decreased persistence in long‐term infections (Brown and Stocker 1987; Sigwart et al. 1989; Saxild and Nygaard 1991; Hoffman et al. 2001; Narayanan et al. 2022; Connolly et al. 2017). Similarly, mutations in *de novo* pyrimidine biosynthesis genes such as *carAB, pyrC, pyrE*, and *pyrF* reduce bacterial fitness in extracellular systemic niches, especially in serum and bloodstream infection models in *E. coli, Bacillus anthracis*, *Acinetobacter baumannii*, and *Shigella flexneri* (Kwaga et al. 1994; Zhuo et al. 2015; Carvalho et al. 2018; Horzempa et al. 2010). Similarly, mutations in *de novo* pyrimidine biosynthesis genes such as *carAB, pyrC, pyrE*, and *pyrF* reduce bacterial fitness in extracellular systemic niches, especially in serum and bloodstream infection models in *E. coli, B. anthracis*, *A. baumannii*, and *S. flexneri* (Kwaga et al. 1994; Zhuo et al. 2015; Carvalho et al. 2018; Horzempa et al. 2010). Mutants of *E. coli* and *S. enterica* that lack genes for purine or pyrimidine synthesis, for example, were incapable of replication in bacteremia models and in human serum (Morgan et al. 2004; Samant et al. 2008). Similarly, *M. tuberculosis* and *S. pneumoniae* are dependent on *de novo* pathways for persistence and growth in macrophages and during systemic infection (Rengarajan et al. 2005; Polissi et al. 1998; Liu et al. 2021). These studies confirm that for infections of nucleoside‐poor niches such as blood, serum, and the CNS, the salvage processes alone are not sufficient to enable survival of bacterial pathogens at the site of infection (Table 1).

In contrast, the relatively high intracellular concentrations of purine and pyrimidine metabolites enable bacteria to survive using salvage pathways to acquire nucleotides (Traut 1994; Mollenkopf et al. 2001; Shaffer et al. 2017; Ackland et al. 2021; Petit and Lebreton 2022; Holmes et al. 2023). For instance, in a mouse model of listeriosis, *L. monocytogenes* mutants lacking the *de novo* pyrimidine biosynthesis genes (*purH*, *pyrE, pyrF*, or *pyrC*) were as virulent as the wild‐type, and *S. enterica ΔpyrE* mutants also exhibited no attenuation in C57BL/6 J mice intestinal colonization model and ileal loop infections in calves, indicating that host‐derived nucleotides supported pathogen growth (Klarsfeld et al. 1994; Schauer et al. 2010; Harvey et al. 2011; Marquis et al. 1993). These studies show that niche‐specific switching of nucleobase acquisition occurs in *S. enterica*, and this has also been demonstrated for *K. pneumoniae*, which relies on *de novo* purine biosynthesis in the blood and lung but not in the liver or spleen, where exogenous purines are accessible. While overall nucleotide availability in the host cytosol is high and many intracellular pathogens are able to capitalize on this (Petit and Lebreton 2022; Steighardt et al. 2000; Fields et al. 1986; Marquis et al. 1993; Traut 1994), the cytosol is not a homogeneous compartment and contains many organelles, in which purine and pyrimidine availability may be limited (Petit and Lebreton 2022; Goetz et al. 2001). Following uptake into the cell, bacteria frequently reside inside membrane‐bound vacuoles that may be nutrient‐poor (Leung and Finlay 1991; Steighardt et al. 2000; Munoz‐Elias and McKinney 2006). An extreme example of such an environment is the macrophage phagosome after invasion, which in many bacterial pathogens, including *M. tuberculosis*, *F. tularensis*, *L. monocytogenes, S. typhimurium, Y. pestis*, and *H. influenzae*, triggers the *de novo* biosynthesis of purines and pyrimidines (Drazek et al. 1995; Horzempa et al. 2010; Munier‐Lehmann et al. 2003; Mahan et al. 1993; Leung and Finlay 1991; Straley and Harmon 1984; Kappler et al. 2024; Ackland et al. 2021). An upregulation of *de novo* nucleotide biosynthesis genes, notably purine biosynthesis, has also been observed for infections of epithelial cells, and mutations in associated genes result in decreased colonization and pathogenicity (Hosmer et al. 2022; Euba et al. 2023; Kappler et al. 2024).

Despite the clear advantages associated with switching between *de novo* synthesis and salvage pathways for nucleobase acquisition during host colonization, several pathogenic bacteria lack *de novo* synthesis or salvage pathways for purines, pyrimidines, or both purines and pyrimidines (Table 1). This is particularly prevalent in bacteria that inhabit environmental niches with high availability of purines and pyrimidines, and has been proposed as a trait linked to adaptation to intracellular lifestyles (Fuchs et al. 2012; Wheeler 1989; Mitchell and Finch 1977) (Chua and Fraser 2020). In keeping with this, several host‐adapted, obligate intracellular pathogens, *Mycoplasma* (Wang et al. 2001), *Ureaplasma* (Wang et al. 2001), *Chlamydia* (Zomorodipour and Andersson 1999), and *Rickettsia* (Zomorodipour and Andersson 1999), lack all genes for *de novo* nucleotide synthesis and depend exclusively on exogenous purine and/or pyrimidine sources for growth. *Mycoplasma* and *Ureaplasma* thrive in the host cytoplasm, while *Chlamydia* reside in modified vacuoles that support their survival and access to nucleoside sources (Benedetti et al. 2020; Waites et al. 2012; Zomorodipour and Andersson 1999; Herweg and Rudel 2016, Bastidas et al.). This highly adapted lifestyle can be supported by an increased number of cellular nucleotide transporters, as has been shown for several bacteria belonging to the Mycoplasmatales (Gelgie et al. 2024; Mitchell and Finch 1977; Wang et al. 2001; Sun and Wang 2013). As would be expected, none of these obligate intracellular bacteria is able to thrive in macrophages where access to nucleosides and nucleobases is highly restricted (Bechelli et al. 2021; Herweg and Rudel 2016; Curto et al. 2019; Reyes et al. 2009; Nolan et al. 2016; Schimmelpfeng et al. 1980). Loss of *de novo* purine and pyrimidine synthesis pathways as an adaptation to an intracellular lifestyle also extends to eukaryotic organisms. Here, parasitic microsporidia, which replicate and form spores during intracellular infection, lack both *de novo* synthesis pathways and also contain an increased number of transporter proteins from different protein families to ensure nucleotide transport (Dean et al. 2016).

Interestingly, some host‐adapted bacterial pathogens show loss of only one of the two *de novo* biosynthesis pathways (Table 1). *Helicobacter pylori*, which colonizes the nutrient‐rich stomach mucosa, lacks the *de novo* purine synthesis machinery and thus depends on purine salvage, presumably owing to the abundance of purine sources in the mucosa (Liechti and Goldberg 2012). *H. pylori* encodes an efficient salvage pathway as observed by the growth defects caused by deletion of purine salvage genes (*gpt, apt*, and *deoD*), which were rescued by addition of exogenous purines that could be acquired by the remaining salvage pathway elements (Liechti and Goldberg 2012). Another study found that *H. pylori* depends on a NupC‐related transporter (HP1180) for purine nucleoside uptake, and that its deletion resulted in poor growth on purines as a sole nucleoside source as well as lower uptake of radiolabelled adenosine, guanosine, and inosine (Miller et al. 2012; Liechti and Goldberg 2013). Deletion of the *guaA* gene that encodes GMP synthase had no effect on mouse stomach colonization, indicating sufficient supply of a variety of purines in this niche. Similarly, the respiratory pathogen *H. influenzae*, which colonizes predominantly the nasopharyngeal mucosa but also thrives in the lower respiratory tract and the middle ear (Paul T King 2015; Duell et al. 2016; Bakaletz and Novotny 2018), lacks three enzymes for the early stage of *de novo* pyrimidine biosynthesis. Despite this, *H. influenzae* can survive not only in mucosal, but surprisingly, also in pyrimidine‐restricted settings, such as CSF (Sinha et al. 2013, Roman L. Tatusov et al. 1996; Mason et al. 2003; Gawronski et al. 2009; Dhouib et al. 2015; Dhouib et al. 2016). At present, it is unclear how *H. influenzae* strains access pyrimidines during CSF colonization, and we propose that this could be linked to the availability of intermediates of pyrimidine biosynthesis that could support growth of *H. influenzae* in pyrimidine‐poor niches are unclear. *De novo* purine biosynthesis is also absent in several non‐pathogenic bacteria, such as *Lactobacillus* species, as a consequence of long‐term adaptation to nutrient‐rich habitats, such as the gut and fermented foods (Kilstrup et al. 2005), confirming the connection between adaptation to nutrient‐rich environments and loss of biosynthetic abilities.

**Figure 4:**
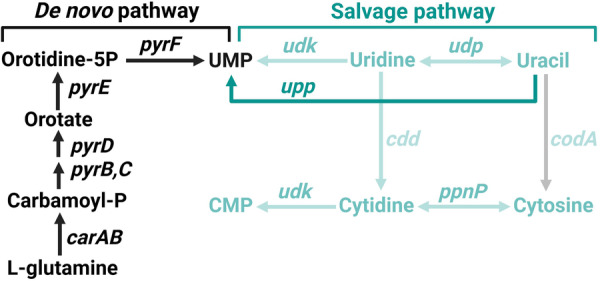
Representation of the pyrimidine *de novo* and salvage pathway in *Mycobacterium* species and *Neisseria meningitidis*. The picture depicts the pyrimidine salvage pathway (blue) and the *de novo* biosynthesis pathway (black) in both species. In contrast to the full *de novo* biosynthesis, the absence of most salvage enzymes is highlighted by the light blue indication of enzymes and reactions that are not present in these bacteria.

Interestingly, a very small number of bacterial pathogens rely exclusively on *de novo* synthesis of pyrimidines and lack the salvage pathways. Notably, several species of *Mycobacterium* and *Neisseria meningitidis* are unable to salvage environmental pyrimidine (Figure 4) (Jyssum and Jyssum 1979; Bange et al. 1996; Sassetti and Rubin 2003; Rengarajan et al. 2005). Both of these pathogens specialize in surviving in environments in which host‐derived purine and pyrimidine sources are scarce, such as alveolar macrophages in the case of *M. tuberculosis*, and the bloodstream and CSF for *N. meningitidis*. This exclusive reliance on *de novo* synthesis of nucleobases in these species reflects their preferred growth niches and is also consistent with research on *E. coli*, which showed that intact *de novo* pathways were necessary for intracellular survival in macrophages and in nutrient‐deficient blood and CSF (Shaffer et al. 2017; Samant et al. 2008). In summary, while purine and pyrimidine availability is a prerequisite for the health and survival of all living cells, in bacteria, specific adaptations to preferred growth niches have led to a loss of either the near ubiquitously present *de novo* biosynthesis and salvage pathways for these compounds that, in many other bacterial species, enable colonization of a wide variety of host niches. The wider evolutionary drivers of these adaptations are only partially known, and further work could shed light, especially on unusual adaptations such as the complete loss of the energetically more favourable pathway of nucleobase salvage in species such as the notoriously slow‐growing *Mycobacteria*.

## 4 Therapeutic Opportunities Targeting Nucleotide Metabolism

The often striking impact of mutations in nucleobase/nucleoside metabolism on bacterial virulence and pathogenicity suggests that these pathways and enzymes might have therapeutic potential (Fields PI et al. 1986; Jackson et al. 1996; Taylor et al. 2002; Ralli et al. 2007; Christiansen et al. 2014; Truong et al. 2015). However, a significant hurdle in developing purine or pyrimidine biosynthesis‐specific therapeutics is the universal conservation of the biosynthesis pathways, where human and bacterial enzymes are often closely related. For example, *M. tuberculosis* PurF and human phosphoribosyl pyrophosphate amidotransferase (PPAT) have around 56% homology (Aiba and Mizobuchi 1989; Škerlová et al. 2020). Similar to this, in pyrimidine biosynthesis, human and *E. coli* share approximately 50%−70% of their sequences in carbamoyl‐phosphate synthetase II (CPSII), aspartate transcarbamoylase (ATCase), and dihydroorotase (DHOase) (Grande‐García et al. 2014; Huang 2025; Evans and Guy 2004). Where differences in enzymes exist, it is often due to the fusion of relevant genes in some species. An example of this is the conversion of AIR to CAIR, where bacteria use individual PurK and PurE proteins, while humans utilize just one bifunctional enzyme (Škerlová et al. 2020; Zhang et al. 2008). The common evolutionary origin of these pathways and the catalytic mechanisms involved make the design of selective inhibitors more challenging. Inhibition of conserved catalytic functions by common antimetabolites such as 5‐fluorouracil, flucytosine (5‐FC) (Chandra and Ghannoum 2017; Vermes 2000), can trigger host toxicity when administered systemically at the necessary levels to eradicate an infection. 5‐FC however differs from 5‐FU where it acts as a prodrug and must first be converted into 5‐fluorouracil by cytosine deaminase (Billmyre et al. 2020). As human cells lack this enzyme, this conversion occurs primarily in fungi and some bacteria, contributing to the more selective toxicity of 5‐FC. The side effects elicited by these drugs include, but are not limited to, hepato‐ and nephrotoxicity, dermatological issues such as rashes and itching, as well as increased risks of cardiac and respiratory failure (Chandra and Ghannoum 2017; Vermes 2000). However, as in most patients, these side effects are dose‐dependent; both 5‐fluorouracil, 5‐FC, and some other drugs that target purine or pyrimidine biosynthesis are in clinical use, usually to target otherwise difficult to eradicate fungal infections, but require strict monitoring of the person undergoing treatment for systemic side effects (Padda and Parmar 2025; Chandra and Ghannoum 2017; Vermes 2000; Schwarz et al. 2006).

The antifungal activity of flucytosine is based on its uptake into the fungal cell, which is followed by intracellular transformation into 5‐fluorouracil by fungal cytosine deaminase (Vermes 2000; Chandra and Ghannoum 2017). 5‐fluorouracil can be incorporated into RNA, causing disruption of RNA synthesis, and additionally inhibits DNA synthesis, resulting in antifungal action (Vermes 2000; Chandra and Ghannoum 2017). With DNA and RNA synthesis targeted, flucytosine and 5‐fluorouracil mostly affect rapidly dividing cells and were, in fact, originally developed as anticancer drugs (Vermes 2000; Rando et al. 2019). Unfortunately, resistance to fluorinated pyrimidine analogues may develop quickly if they are administered alone, requiring combination therapy to maintain effectiveness. As a result, flucytosine is often used in conjunction with amphotericin B, a polyene‐type antifungal, to increase effectiveness and prevent resistance development in the treatment of cryptococcal meningitis (Schwarz et al. 2006). The effectiveness of pyrimidine analogues to eradicate pathogens is not limited to fungi, and treatments with floxuridine, 5‐fluorouracil, or 5‐fluorocytosine have been reported to strongly inhibit proliferation in a range of Gram‐positive and Gram‐negative bacteria, including *E. coli*, *Streptococcus* species, *S. aureus*, and *M. tuberculosis* (Thomson and Lamont 2019; Li et al. 2022; Li et al. 2023; Ravishankar et al. 2024; Martinussen et al. 1994; LOFFLER et al. 2005; King et al. 2006; Niazy et al. 2022). In addition to inhibiting DNA and RNA synthesis, the drugs also inhibit bacterial cell wall biosynthesis, as has been demonstrated for *E. coli*, *S. aureus, P. aeroginosa*, and *M. tuberculosis* (Ravishankar et al. 2024; Zuo et al. 2024; Singh et al. 2015; Ueda et al. 2009; Di Bonaventura et al. 2023).

Other nucleoside analogues developed for chemotherapy have also been investigated for their potential to be used as antimicrobials. The pyrimidine analogues gemcitabine, zidovudine, and floxuridine have all shown varying degrees of selectivity for Gram‐positive and Gram‐negative bacteria (Baldwin et al. 1999; Patching et al. 2005; King et al. 2006; King et al. 2006; Parker and Long 2007; Sandrini et al. 2007; Doléans‐Jordheim et al. 2011; Sun and Wang 2013; Sun and Wang 2013; Yuan et al. 2019). However, all of these drugs affect DNA synthesis, leading to significant side effects in humans, where both gemcitabine and zidovudine are known to cause anaemia by affecting bone marrow function (Malayeri et al. 1997; Sun and Wang 2013; Wang et al. 2014a; Iosifidou et al. 2024; Walker et al. 1988; Keith et al. 1989; Chitnis et al. 2002; Peyclit et al. 2018; Tian et al. 2024). MICs for a range of purine and pyrimidine analogues tested for their effect on pathogenic bacteria showed not only species‐dependent variation, but also varied significantly between strains of the same bacterial species (Sandrini et al. 2007), indicating either low conservation of residues that determine affinity to the drugs on target enzymes, or changes in the efficiency of drug uptake between strains. The purine analogue, zidovudine (also known as 3’‐azido‐3’‐deoxythymidine), was most effective against a range of Gram‐negative bacteria (Doléans‐Jordheim et al. 2011; Tian et al. 2024; Sandrini et al. 2007; Peyclit et al. 2018; Falagas et al. 2019), Conversely, gemcitabine mostly showed antibacterial action against Gram‐positive bacteria, such as *Staphylococcus, Listeria, Enterococcus*, and *Bacillus* species (Sandrini et al. 2007; Sun and Wang 2013; Iosifidou et al. 2024), while floxuridine showed activity against Gram‐negative and Gram‐positive bacteria (Sandrini et al. 2007). In the case of gemcitabine and zidovudine, the drug molecules are phosphorylated and incorporated into the bacterial DNA, causing chain termination during DNA synthesis, while floxuridine is converted to 5‐fluorouracil, which is subsequently metabolised to 5‐fluoro‐2′‐deoxyuridine monophosphate (5‐FdUMP), the active metabolite that inhibits thymidylate synthase (Keith et al. 1989; Wang et al. 2023; Iosifidou et al. 2024; Li et al. 2022; Li et al. 2023).

Despite the promising in vitro activity of these nucleobase and nucleoside analogues, currently none are in clinical use to treat bacterial infections. There is, however, evidence that gemcitabine, administered to patients to treat pancreatic cancer, is in part metabolized by bacteria, including tumor‐associated bacteria (Geller et al. 2017; Corty et al. 2020). This was shown in a study that investigated the association between antibacterial treatments and gemcitabine overdose in cancer patients and found that patients undergoing antibiotic therapy, as well as chemotherapy, were more likely to develop gemcitabine overdose symptoms (Corty et al. 2020). This reveals that during standard chemotherapy, some of the drug is not metabolized by human cells, but by bacteria.

Purine analogues with antimicrobial effects are mostly sulfur‐derivatives of purine nucleobases that are activated by hypoxanthine‐guanine phosphoribosyltransferase and inhibit central enzymes in purine biosynthesis, such as inosine monophosphate dehydrogenase (IMPDH) (Thomson and Lamont 2019). An example is 6‐methylmercaptopurine, which inhibits purine formation, in particular of adenine, and has shown specific toxicity against *Leishmania* species (Azzouz and Lawton 2017). However, these compounds have similar cytotoxicity profiles to the purine nucleobase analogues discussed above, with strong hepatotoxic effects.

Despite the numerous issues with cytotoxicity arising from inhibition of purine or pyrimidine biosynthesis in human cells, recently, some promising new experimental strategies have emerged. JN‐6640 is a structure‐driven and therefore target‐specific inhibitor of PurF in *M. tuberculosis* (Lamprecht et al. 2025). JNJ‐6640 showed in vitro efficiency in the nanomolar range as well as potency in mouse models, where it increased the efficiency of bedaquiline/pretomanid treatments, proving that targeted inhibition of *de novo* purine biosynthesis is achievable. JNJ‐6640 shows low cytotoxicity to human cells, but is currently not considered for clinical use due to its metabolic instability, i.e., its fast degradation rate that makes it unsuitable for oral delivery (Lamprecht et al. 2025). Similarly, *A. baumannii* virulence can be inhibited by specific inhibitors of the *A. baumannii* IMP dehydrogenase (GuaB), which led to a significant improvement in survival in mouse models of bacterial sepsis, with no notable resistance emerging and negligible host toxicity (Peng et al. 2024; Kofoed et al. 2024). The effectiveness of bacteria‐specific inhibitors was already demonstrated in the early 2000s when selective *pyrD* inhibitors eliminated *H. pylori* infections in mice while leaving gut commensals and host cells unaffected, demonstrating the possibility for narrow‐spectrum medicines that minimize microbiome impact (Copeland et al. 2000).

Nucleotide biosynthesis can also be disrupted by interrupting folate‐dependent one‐carbon metabolism. Folate cofactors are required for key steps in purine and thymidylate synthesis, and their depletion leads to reduced nucleotide pools and impaired DNA replication (Li et al. 2017; Goldstein and Proctor 2008; Stover 2009; Yaeger et al. 2025). This vulnerability is exploited by antimicrobial agents such as sulfonamides and trimethoprim, which inhibit folate biosynthesis and reduce the availability of tetrahydrofolate (Bermingham and Derrick 2002). As mammals rely on dietary folate rather than *de novo* synthesis, this pathway provides a selective target for antibacterial therapy (Bourne 2014). To the best of our knowledge, none of these compounds is currently approved for clinical use, but they show promise for future application. The development of specific inhibitors should be expanded to additional bacterial species and also include exploration of inhibitors of bacterial nucleoside and nucleobase transporters.

The concept of inhibition of purine and pyrimidine transport has so far mostly been explored for the treatment of parasitic infection, such as with *Plasmodium falciparum*, the causative agent of malaria (Chen et al. 2025). Bacterial and human nucleoside and nucleobase transporters have little sequence identity. For instance, in humans, two separate transporter families: the sodium‐independent equilibrative nucleoside transporters (ENTs, SLC29) and the sodium‐dependent concentrative nucleoside transporters (CNTs, SLC28) are used to mediate the uptake and efflux of nucleoside and nucleoside‐analog drugs in humans (Young et al. 2013; King et al. 2006). Whereas in bacteria, transport takes place via NCS1/NCS2 and the NHS family (Saxild et al. 1996). Although these families provide comparable physiological functions, they have low sequence homology. As a result, whereas parasite transporter inhibition has produced selective therapeutic leads, bacterial transporter inhibition requires further attention.

Interestingly, externally supplied nucleosides may disrupt bacterial signalling and nucleotide pools, making pathogens more susceptible to traditional antibiotics (Alvarez et al. 2010; Sun and Wang 2013; Yadav et al. 2014; Yuan et al. 2019; Thomson and Lamont 2019). Exogenous provision of purine nucleosides, such as guanosine and xanthosine, decreased c‐di‐AMP levels in MRSA, which in turn reduced MRSA β‐lactam resistance (Li et al. 2018; Li et al. 2021; Nolan et al. 2022; Wang et al. 2023). The ability of the bacteria to absorb and use the nucleosides was necessary to enable this mechanism, as bacteria missing either transporters (*nupG, pbuX*) or enzymes involved in nucleoside salvage (*deoD2, hpt*) did not show the same sensitization effects observed in the wildtype (Nolan et al. 2022). However, how this effect could be exploited in vivo is currently unclear.

Lastly, a very different strategy for application is the use of bacterial strains carrying mutations in *de novo* biosynthesis pathways as live attenuated vaccine strains, a technique that has been demonstrated for several intracellular pathogens such as *B. melitensis*, Salmonella, *M. tuberculosis*, and *F. tularensis* in (Sigwart et al. 1989; Lawrence et al. 1997; Jackson et al. 1999; Quarry et al. 2007; Shirey et al. 2008; Truong et al. 2015). However, given the known risks of live attenuated vaccine strains, significant exploration of the risks of reversion of the attenuated strains to be able to cause disease will be required. *De novo* biosynthesis mutants have been shown to be effective as live‐attenuated vaccines in a number of studies, but none of them have yet to be approved for use in either humans or animals. While the evolutionary conservation of purine and pyrimidine biosynthesis and the resulting cross‐reactivity with human enzymes is a major hurdle for exploiting these processes to treat bacterial infections, recent advances in the development of bacteria‐specific inhibitors are a major step forward in realizing the therapeutic potential of these essential pathways.

## 5 Conclusions

Despite the universal conservation of pathways for the *de novo* synthesis of salvage of purine and pyrimidine nucleobases, nucleosides, and nucleotides, the current literature shows that in bacteria, significant variation in the presence of these pathways exists (Garavaglia et al. 2012; Nyhan 2014; Chua and Fraser 2020; Goncheva et al. 2022; Ayoub et al. 2024). The main driver for the loss of either *de novo* or salvage pathways for either purines or pyrimidines appears to be their availability in the preferred environmental niches that bacteria inhabit (Fields PI et al. 1986; Jackson et al. 1996; Taylor et al. 2002; Ralli et al. 2007; Christiansen et al. 2014; Truong et al. 2015; Connolly et al. 2017). Bacteria with strong niche adaptation are most likely to show loss of function in some pathways, which in some cases, such as in *Mycoplasma*, *Chlamydia*, but also bacteria such as *H. influenzae*, is accompanied by a reduction in genome size (Gawronski et al. 2009; Herweg and Rudel 2016). Bacterial pathogens that contain fully functional *de novo* synthesis and salvage pathways for both purines and pyrimidines are generally able to colonize a wider range of environmental niches and switch between predominant use of *de novo* synthesis and salvage pathways to match environmental availability of purines and pyrimidines.

To realize the potential of nucleobase acquisition and biosynthesis as potential Achilles heels of pathogenic bacteria, bacteria‐specific, and potentially even organism‐specific inhibitors appear to be the most promising route to avoid extensive side effects in the host organism as well as the general microbiome. Possible targets could include transport proteins that are essential for nucleobase salvage; however, the effectiveness of such drugs might be limited to treatment of infections caused by niche‐adapted bacteria, or for the treatment of infections in specific niches for bacteria able to switch between *de novo* biosynthesis and salvage pathways. Where specific inhibitors are available, their ability to resensitize bacteria to commonly used antimicrobials should be investigated. Further studies will be needed to fully understand the links between pathogen evolution and nucleobase metabolism during infection to fully identify the potential role of these pathways and to understand their role in bacterial physiology during interactions with host cells.

## Author Contributions

**Riya Joshi:** conceptualization, investigation, writing – original draft, writing – review and editing, visualization, data curation, validation. **Alastair G. McEwan:** writing – review and editing, funding acquisition. **Ulrike Kappler:** conceptualization, writing – original draft, writing – review and editing, validation, funding acquisition, supervision, visualization.

## Ethics Statement

The authors have nothing to report.

## Conflicts of Interest

The authors declare no conflicts of interest.

## Data Availability

Data sharing not applicable to this article as no datasets were generated or analyzed during the current study.
